# Tonic Neuromodulation of the Inspiratory Rhythm Generator

**DOI:** 10.3389/fphys.2012.00253

**Published:** 2012-07-20

**Authors:** Fernando Peña-Ortega

**Affiliations:** ^1^Departamento de Neurobiología del Desarrollo y Neurofisiología, Instituto de Neurobiología, UNAM-Campus JuriquillaQuerétaro, Mexico

**Keywords:** endogenous neuromodulation, glia, network activity, pacemaker neurons, reconfiguration, extrasynaptic

## Abstract

The generation of neural network dynamics relies on the interactions between the intrinsic and synaptic properties of their neural components. Moreover, neuromodulators allow networks to change these properties and adjust their activity to specific challenges. Endogenous continuous (“tonic”) neuromodulation can regulate and sometimes be indispensible for networks to produce basal activity. This seems to be the case for the inspiratory rhythm generator located in the pre-Bötzinger complex (preBötC). This neural network is necessary and sufficient for generating inspiratory rhythms. The preBötC produces normal respiratory activity (eupnea) as well as sighs under normoxic conditions, and it generates gasping under hypoxic conditions after a reconfiguration process. The reconfiguration leading to gasping generation involves changes of synaptic and intrinsic properties that can be mediated by several neuromodulators. Over the past years, it has been shown that endogenous continuous neuromodulation of the preBötC may involve the continuous action of amines and peptides on extrasynaptic receptors. I will summarize the findings supporting the role of endogenous continuous neuromodulation in the generation and regulation of different inspiratory rhythms, exploring the possibility that these neuromodulatory actions involve extrasynaptic receptors along with evidence of glial modulation of preBötC activity.

## Introduction

Neural network activity relies on the interactions between intrinsic and synaptic properties (Ramirez et al., 2004; Marder and Bucher, 2007; Peña, 2009). Here, I will consider “synaptic properties” as those provided by fast neurotransmission among neurons and “neuromodulation” as the slower changes in cellular and synaptic properties mediated by metabotropic receptors (Katz, 1998). Neuromodulators regulate the activity of networks, allowing their adaptation to different demands or even conditioning their basal activity (Katz, 1998; Tryba et al., 2006; Peña, 2009). This issue has been studied in the inspiratory rhythm generator, the pre-Bötzinger complex (preBötC), which generates the inspiratory commands that control the diaphragm (Smith et al., 1991; Feldman and Del Negro, 2006; Schwarzacher et al., 2011). Breathing is eliminated by lesioning or inactivating the preBötC (Ramirez et al., 1998; Wenninger et al., 2004), whereas the isolated preBötC is still able to generate the inspiratory rhythms in a brainstem slice preparation (Smith et al., 1991; Lieske et al., 2000; Peña et al., 2008; Armstrong et al., 2010) or even in preBötC islands (Ramírez-Jarquín et al., 2012).

In slices, the preBötC generates three distinct activity patterns that correspond to distinct forms of breathing: normal respiratory activity (eupnea), sighs, and gasps (Lieske et al., 2000). Gasping is generated during hypoxia as a “last-resort” respiratory effort to autoresuscitate (Gozal et al., 2002; Fewell et al., 2007; Zavala-Tecuapetla et al., 2008). Interestingly, babies that die from SIDS have a reduction in gasping generation and inefficient autoresuscitation (Poets et al., 1999; Sridhar et al., 2003). The preBötC contains several types of neurons, including expiratory, inspiratory, and postinspiratory neurons (Lieske et al., 2000) that interact through fast synaptic transmission (Greer et al., 1991; Funk et al., 1993; Shao and Feldman, 1997; Ren and Greer, 2006) to produce the inspiratory rhythms. Among the inspiratory preBötC neurons, a group of respiratory pacemaker neurons has been detected that plays a major role in rhythm generation (Thoby-Brisson and Ramirez, 2001; Peña et al., 2004; Del Negro et al., 2005; Peña and Aguileta, 2007). Characterization of these neurons revealed at least two types (Thoby-Brisson and Ramirez, 2001; Peña et al., 2004; Del Negro et al., 2005; Peña and Aguileta, 2007; Peña, 2008), one that generates bursts via a Ca^2+^-activated cationic current (*I*_CAN_) and the other that relies on the persistent Na^+^ current (*I*_NaP_; Peña et al., 2004; Del Negro et al., 2005; Peña and Aguileta, 2007; Peña, 2008). Both types of pacemakers need to be inhibited to abolish rhythmogenesis under normoxia, both *in vitro* and *in vivo* (Peña et al., 2004; Peña and Ramirez, 2005; Tryba et al., 2006). In contrast, during hypoxic conditions, gasping generation critically relies on the activity of *I*_NaP_-dependent (hypoxia-resistant) pacemaker neurons (Peña et al., 2004; Tryba et al., 2006; Peña and Aguileta, 2007). In addition to these mechanisms, the specific contribution of intrinsic and synaptic properties to rhythmogenesis depends on the neuromodulatory context. When applied exogenously, several neuromodulators modify the generation of the rhythmic activity by the preBötC (Doi and Ramirez, 2008). Moreover, several of these neuromodulators maintain a continuous endogenous modulation that, in some cases, is indispensable for rhythm generation (Peña and Ramirez, 2002, 2004; Tryba et al., 2006; Viemari et al., 2011; Ramírez-Jarquín et al., 2012). This continuous modulation, synonymous with “tonic neuromodulation,” is maintained by continuous release of neuromodulators, mainly from tonic active neurons and glial cells (Hülsmann et al., 2000; Ptak et al., 2009).

### Continuous (“tonic”) neuromodulation of the preBötC

The actions of different neuromodulators on the inspiratory rhythm generator, including amines and peptides, were recently reviewed (Ballanyi, 2004; Peña and García, 2006; Doi and Ramirez, 2008; Peña, 2009). Therefore, I will focus on the evidence of endogenous continuous neuromodulations of the preBötC. In the CNS, several neuromodulators can be continuously released and act both at synaptic and extrasynaptic levels to regulate network function (Vizi et al., 2010). Extrasynaptic transmission was originally discovered for several monoamines that regulate the release of other neuromodulators and neurotransmitters despite the lack of synaptic contact between the two terminals (Vizi et al., 2010). In fact, the majority of monoaminergic and peptidergic neurons fail to make synaptic contacts and instead, they act on extrasynaptic receptors (Descarries and Mechawar, 2000; Vizi et al., 2010). Such neuromodulators are preferentially, but not exclusively, accumulated in large, dense-core vesicles, and they require a strong depolarization or high frequency stimulation to be released (Torrealba and Carrasco, 2004; De-Miguel and Trueta, 2005; Vizi et al., 2010). The fact that several neuromodulators, such as serotonin and adenosine, have been detected in the extracellular space of the preBötC by means of microdialysis (Richter et al., 1999), which detects neurotransmitters and neuromodulators that escaped the synaptic cleft (Peña and Tapia, 1999, 2000), suggests that they can reach extrasynaptic receptors and continuously modulate the preBötC. The extracellular concentration of these neuromodulators changes depending on the state of the network (i.e., hypoxia; Richter et al., 1999; Hehre et al., 2008) indicating that such continuous modulation adjusts the preBötC activity to fit particular demands. Next, I will present a catalog of neuromodulators that maintain a continuous neuromodulation of the preBötC, and discuss the possible involvement of extrasynaptic receptors or glial cells in this modulation. It is important to consider that respiratory rhythmogenesis is studied in a variety of experimental conditions ranging from behaving animals to preBötC islands (Ramírez-Jarquín et al., 2012). Thus, in most cases, the pharmacological manipulations could affect different respiratory circuits besides the preBötC (Zavala-Tecuapetla et al., 2008; Ramírez-Jarquín et al., 2012).

### Adenosine

Adenosine is an inhibitory neuromodulator of the preBötC (Schmidt et al., 1995; Herlenius and Lagercrantz, 1999; Wilken et al., 2000; Huxtable et al., 2009) that can be directly released from neurons and glia or that can be extracellularly produced by the degradation of released ATP (Martín et al., 2007; Cunha, 2008; Zwicker et al., 2011). Ambient adenosine can exert its effects by diffusing far away from the release sites (Cunha, 2008; Vizi et al., 2010). An adenosinergic continuous modulation of the preBötC of mice has been evidenced by blocking adenosine-receptors with the non-selective, adenosine-receptor antagonist aminophylline (Wilken et al., 2000), which increases the frequency and amplitude of inspiratory rhythm in slices. This effect is similar to blocking the type 1 (A1) adenosine-receptor in rats with the specific antagonist DPCPX (Huxtable et al., 2009) These increases have also been observed in the brainstem-spinal cord preparation (also called the “*en bloc*”) of rats (Herlenius and Lagercrantz, 1999) and in cats *in vivo* (Schmidt et al., 1995), where levels of adenosine increase in hypoxia (Richter et al., 1999), contributing to the respiratory depression observed during this condition. In fact, blocking A1-receptors attenuates hypoxia-induced breathing in the *en bloc* of rats (Kawai et al., 1995). Thus, it has been suggested that adenosine antagonists can be useful for the treatment of several respiratory dysfunctions (Mathew, 2011).

### ATP

ATP excites the preBötC *in vitro* in rats (Huxtable et al., 2009; Zwicker et al., 2011) through the activation of P2Y-receptors (Lorier et al., 2007; Huxtable et al., 2009). Interestingly, blockade of endogenous activation of P2-receptors with suramin reduced inspiratory frequency in the slice preparation, while Cu^2+^, an allosteric modulator of purinergic receptors, produced the opposite effect (Lorier et al., 2007, 2008). ATP is released during hypoxia, and blocking its tonic action on P2-receptors increases the hypoxia-induced slowing of the respiratory rhythm, suggesting that ATP is involved in maintaining respiration in hypoxia in rats (Gourine et al., 2005). Interestingly, the excitatory effect of exogenous ATP on the preBötC is precluded when glial cells are inhibited (Huxtable et al., 2009).

### Acetylcholine

Acetylcholine (ACh) is another neuromodulator that tonically regulates preBötC activity in rats and mice (Shao and Feldman, 2009). Application of the acetylcholinesterase inhibitor physostigmine increases the frequency of rhythmic respiratory activity in the slice preparation involving the type-3-muscarinic and α4β2-nicotinic receptors in rats and mice, respectively (Shao and Feldman, 2005; Shao et al., 2008). Similarly, blockade of muscarinic-receptors with atropine reduces the amplitude and frequency of the respiratory rhythm in the *en bloc* from mice (Coddou et al., 2009). In the lamprey *en bloc*, physostigmine increases the respiratory frequency, while the nicotinic antagonists D-tubocurarine or bungarotoxin reduces it (Mutolo et al., 2011).

### Noradrenaline

Pre-Bötzinger complex activity is modulated by endogenous noradrenaline released from the A5, A6, A1C1, and A2C2 nuclei in rats and mice (Hilaire et al., 2004; Viemari, 2008). This continuous modulation involves activation of α-2-adrenoreceptors, since its blockade with yohimbine, piperoxane, or phentolamine decreases respiratory frequency in the *en bloc* in rats and mice (Errchidi et al., 1990; Zanella et al., 2006; Fujii and Arata, 2010) and abolishes gasping generation in slices from mice (Viemari et al., 2011). Accordingly, decreasing the extracellular noradrenaline concentration with pargyline, desipramine, or tyrosine increases the frequency of the rhythm, while methyltyrosine, an inhibitor of noradrenaline biosynthesis, increases the *en bloc* respiratory frequency in rats and mice (Errchidi et al., 1990; Zanella et al., 2006). There is some evidence of a continuous modulation of the preBötC by histamine and dopamine. Thus, the histamine-type-1-receptor antagonist, pyrilamine, reduces the *en bloc* respiratory frequency and attenuates respiratory depression in hypoxia in mice (Dutschmann et al., 2003), while the dopamine-type-1-receptor antagonist SCH-23390 slows the respiratory rhythm of cats *in vivo* (Lalley, 2004, 2005).

### Serotonin

The preBötC is modulated by 5-hydroxytryptamine (5-HT), which produces an excitatory effect mediated by 5-HT2-receptors and an inhibitory effect mediated by 5-HT1-receptors (Schwarzacher et al., 2002). The main source of 5-HT is the raphe nuclei (Richerson, 2004), whose projections can or cannot make synaptic contacts with their targets throughout the brain (Kosofsky and Molliver, 1987). In the preBötC, increasing the extracellular concentration of 5-HT with 5-HT-uptake inhibitors leads to an increase of respiratory activity in the *en bloc* from rats (Di Pasquale et al., 1994). In contrast, blocking 5-HT-receptors with the non-specific antagonist methysergide abolishes rhythmogenesis in the *en bloc* and in slices from rats (Di Pasquale et al., 1994; Ptak et al., 2009). In these preparations, excitation of raphe neurons increases the frequency of the respiratory rhythm mediated by the activation of 5-HT2-receptors (Al-Zubaidy et al., 1996; Ptak et al., 2009). Accordingly, blocking either 5-HT2B-receptors (Günther et al., 2006), 5-HT2C-receptors (Ptak et al., 2009), or 5-HT2A receptors (Peña and Ramirez, 2002; Ptak et al., 2009) reduces the respiratory rhythm frequency and its regularity in slices from rats and mice. Such findings have been corroborated for 5-HT2A- and 5-HT2C-receptors *in situ* in rats (Ptak et al., 2009). Interestingly, low micromolar concentrations of 5-HT induce bursting activity in non-bursting preBötC neurons (Ptak et al., 2009), while blockade of 5-HT2A receptors abolishes the intrinsic bursting of the *I_NaP_*-dependent (hypoxia-resistant) pacemaker neurons (Peña and Ramirez, 2002; Tryba et al., 2006). Consequently, blockade of 5-HT2A receptors inhibits gasping generation in slices from mice (Tryba et al., 2006) and *in situ* in rats (Bale and Solomon, 2010). These findings may have clinical relevance, since it has been hypothesized that a deficiency of the medullary 5-HT network is a potential cause of SIDS (Kinney et al., 2001).

### Peptides

Several neuropeptides may exert a continuous regulation of the preBötC. Neuropeptides are typical non-synaptic transmitters, which are released extrasynaptically (Torrealba and Carrasco, 2004; Wotjak et al., 2008). Blocking the endogenous activation of the opioid-receptors with naloxone increases the respiratory output in cats (Lawson et al., 1979) and reduces hypoxia-induced respiratory depression in rats (Schlenker and Inamdar, 1995). In mice, blocking endogenous activation of somatostatin-receptors increases the respiratory rhythm frequency and reduces its regularity, both in slices and *in vivo* (Ramírez-Jarquín et al., 2012). Moreover, blockade of somatostatin-receptors, specifically subtype 2, prevents the reconfiguration of the preBötC during hypoxia *in vitro* and reduces gasping generation and autoresuscitation *in vivo* (Ramírez-Jarquín et al., 2012). In contrast, substance-P maintains an excitatory continuous modulation on the preBötC in rats and mice (Ptak et al., 2009; Doi and Ramirez, 2010). Blockade of the substance-P receptor (NK1) with SR 140333 or spantide inhibits rhythmogenesis *in vitro* and *in situ* in mice and rats, respectively (Telgkamp et al., 2002; Ptak et al., 2009). Interestingly, in mice, inhibition of respiratory activity with NK1 antagonists has no significant respiratory effect when the levels of 5-HT or noradrenaline are increased by stimulating the raphe magnus or locus coeruleus, respectively (Doi and Ramirez, 2010), indicating that the action of substance-P might be influenced by the neuromodulatory state of the network (Doi and Ramirez, 2010).

### Possible regulation of the preBötC by GABA and glutamate acting on extrasynaptic receptors

Glutamatergic and GABAergic neurons were thought to release their transmitters exclusively at synapses, where they mediate the classical “fast synaptic transmission” (Vizi et al., 2010). However, it has been shown that ambient GABA and glutamate can also tonically activate high-affinity, extrasynaptic receptors, suggesting their spill-over from synaptic boutons, mediating a slower synaptic transmission (Semyanov et al., 2004; Farrant and Nusser, 2005; Aghajanian, 2009). Extrasynaptic GABA_A_ inhibition can modulate the generation of hippocampal fast rhythms (Scanziani, 2000; Towers et al., 2004; Mann and Mody, 2010; Papatheodoropoulos and Koniaris, 2011), and it is likely that such modulation also occurs in the preBötC, where increasing the extracellular concentration of GABA, by inhibiting its uptake with nipecotic acid, decreases the respiratory frequency (Ren and Greer, 2006). The presence of delta-subunit-containing-GABA_A_-receptors, which are mainly extrasynaptic (Nusser et al., 1998; Adkins et al., 2001; Brown et al., 2002) suggests a tonic GABAergic control of the preBötC. For instance, the application of the GABA_A_-receptor agonist THIP, which preferentially activates extrasynaptic GABA_A_-receptors containing delta-subunits (Nusser et al., 1998; Adkins et al., 2001; Brown et al., 2002), hyperpolarizes respiratory neurons and reduces the frequency of the respiratory rhythm (Shao and Feldman, 1997). Neurosteroids, which also target delta-containing, extrasynaptic GABA_A_-receptors (Stell et al., 2003; Belelli and Lambert, 2005; Scimemi et al., 2006), modulate GABA_A_-receptor-mediated hyperpolarization of respiratory neurons and the inhibition of rhythmogenesis in slices (Ren and Greer, 2006).

Ambient glutamate can also activate extrasynaptic, NR2B-subunit-containing, NMDA-receptors and modulate neural network activity (Lambe and Aghajanian, 2006, 2007; Aghajanian, 2009). It is likely that extrasynaptic, NMDA-receptor-mediated excitation is also present in the preBötC, where inhibition of glutamate uptake with dihydrokainate increases rhythmogenesis (Greer et al., 1991; Funk et al., 1993). Dihydrokainate can also restore rhythmogenesis in substance-P-depleted slices, in which capsaicin abolishes rhythm generation (Morgado-Valle and Feldman, 2004). Similarly, releasing NMDA-receptors from their Mg^2+^-blockade restores rhythmogenesis in slices where the rhythm is abolished by AMPA-receptor blockade (Morgado-Valle and Feldman, 2007). This evidence supports the notion that a tone of extracellular glutamate can participate in rhythmogenesis. Furthermore, the presence of the NR2B-receptor has been extensively documented in the preBötC (Watanabe et al., 1994; Paarmann et al., 2000, 2005; Liu and Wong-Riley, 2010).

### Glial modulation of the preBötC

Glial cells are integral functional elements of neural networks, since it is argued that they can respond to and regulate neuronal activity (Araque and Navarrete, 2010). The respiratory network is not an exception (Gourine et al., 2010). Glial cells can sense preBötC activity, and a portion of them display a phase-locked rhythmic activity (Schnell et al., 2011). Moreover, glial cells are essential for rhythmogenesis, since both fluoroacetate, which selectively blocks the glial Krebs cycle, and methionine-sulfoximine, which blocks glutamine synthetase (Hülsmann et al., 2000; Young et al., 2005; Huxtable et al., 2010), inhibit rhythmic respiratory burst activity in slices. In these conditions, addition of isocitrate or glutamine restores the rhythmic network activity (Hülsmann et al., 2000). Accordingly, methionine-sulfoximine-treated pups displayed a reduced breathing frequency and a reduced responsiveness to hypercapnia (Young et al., 2005). Moreover, glial cells are required not only for maintaining rhythm generation but also for the response of the preBötC to neuromodulators or to metabolic demands (Gourine et al., 2010). For instance, fluoroacetate and methionine-sulfoximine reduce preBötC responsiveness to ATP (Huxtable et al., 2010), and preBötC glial cells can respond to preBötC neuromodulators including 5-HT and substance-P (Härtel et al., 2009).

I conclude that continuous neuromodulation exerts a powerful influence on the preBötC; to the extent that, in some cases, it is necessary for rhythm generation. Continuous neuromodulation tunes the excitability of the preBötC to respond to different demands and also determines the weight of specific neuronal types or specific synaptic interactions in the generation of network dynamics. This property could allow the preBötC to adopt an infinite number of conformations based on the same circuit (neural units and connections). Moreover, the evidence that one neuromodulation is determined by tonic control exerted by other neuromodulators, supports the notion that the intrinsic and synaptic properties of the preBötC are not fixed, but can change in a state-dependent manner. The levels of modulation in the preBötC would determine the availability of neural properties (intrinsic, synaptic, or both) that can participate in network dynamics or are susceptible to subsequent neuromodulation.

## Conflict of Interest Statement

The author declares that the research was conducted in the absence of any commercial or financial relationships that could be construed as a potential conflict of interest.

## References

[B1] Adkins C. E. Pillai G. V. Kerby J. Bonnert T. P. Haldon C. McKernan R. M. Gonzalez J. E. Oades K. Whiting P. J. Simpson P. B. (2001). Alpha4beta3delta GABAA receptors characterized by fluorescence resonance energy transfer-derived measurements of membrane potential. J. Biol. Chem. 276, 38934–38939 10.1074/jbc.M104318200 11495904

[B2] Aghajanian G. K. (2009). Modeling “psychosis” in vitro by inducing disordered neuronal network activity in cortical brain slices. Psychopharmacology (Berl.) 206, 575–585 10.1007/s00213-009-1484-9 19241062 PMC2755104

[B3] Al-Zubaidy Z. A. Erickson R. L. Greer J. J. (1996). Serotonergic and noradrenergic effects on respiratory neural discharge in the medullary slice preparation of neonatal rats. Pflugers Arch. 431, 942–949 10.1007/BF02332181 8927513

[B4] Araque A. Navarrete M. (2010). Glial cells in neuronal network function. Philos. Trans. R. Soc. Lond. B. Biol. Sci. 365, 2375–2381 10.1098/rstb.2009.0313 20603358 PMC2894949

[B5] Armstrong G. A. López-Guerrero J. J. Dawson-Scully K. Peña F. Robertson R. M. (2010). Inhibition of protein kinase G activity protects neonatal mouse respiratory network from hyperthermic and hypoxic stress. Brain Res. 1311, 64–72 10.1016/j.brainres.2009.11.038 19945442

[B6] Bale T. A. Solomon I. C. (2010). Influence of 5-HT2A receptor blockade on phrenic nerve discharge at three levels of extracellular K+ in arterially-perfused adult rat. Adv. Exp. Med. Biol. 669, 139–142 10.1007/978-1-4419-5692-7_28 20217337

[B7] Ballanyi K. (2004). Neuromodulation of the perinatal respiratory network. Curr. Neuropharmacol. 2, 221–243 10.2174/1570159043476828 30509012

[B8] Belelli D. Lambert J. J. (2005). Neurosteroids, endogenous regulators of the GABAA receptor. Nat. Rev. Neurosci. 7, 565–575 10.1038/nrn1703 15959466

[B9] Brown N. Kerby J. Bonnert T. P. Whiting P. J. Wafford K. A. (2002). Pharmacological characterization of a novel cell line expressing human alpha 4beta 3delta GABAA. receptors. Br. J. Pharmacol. 136, 965–974 10.1038/sj.bjp.0704795 12145096 PMC1573424

[B10] Coddou C. Bravo E. Eugenín J. (2009). Alterations in cholinergic sensitivity of respiratory neurons induced by pre-natal nicotine, a mechanism for respiratory dysfunction in neonatal mice. Philos. Trans. R. Soc. Lond. B. Biol. Sci. 364, 2527–2535 10.1098/rstb.2009.0078 19651654 PMC2865120

[B11] Cunha R. A. (2008). Different cellular sources and different roles of adenosine, A1 receptor-mediated inhibition through astrocytic-driven volume transmission and synapse-restricted A2A receptor-mediated facilitation of plasticity. Neurochem. Int. 52, 65–72 10.1016/j.neuint.2007.06.026 17664029

[B12] Del Negro C. A. Morgado-Valle C. Hayes J. A. Mackay D. D. Pace R. W. Crowder E. A. Feldman J. L. (2005). Sodium and calcium current-mediated pacemaker neurons and rhythmogenesis. J. Neurosci. 25, 446–453 10.1523/JNEUROSCI.2237-04.2005 15647488 PMC6725489

[B13] De-Miguel F. F. Trueta C. (2005). Synaptic and extrasynaptic secretion of serotonin. Cell. Mol. Neurobiol. 25, 297–312 10.1007/s10571-005-3061-z 16047543 PMC11529639

[B14] Descarries L. Mechawar N. (2000). Ultrastructural evidence for diffuse transmission by monoamine and acetylcholine neurons of the central nervous system. Prog. Brain Res. 125, 27–47 10.1016/S0079-6123(00)25005-X 11098652

[B15] Di Pasquale E. Monteau R. Hilaire G. (1994). Endogenous serotonin modulates the fetal respiratory rhythm, an in vitro study in the rat. Brain Res. Dev. Brain Res. 80, 222–232 10.1016/0165-3806(94)90107-4 7955347

[B16] Doi A. Ramirez J. M. (2008). Neuromodulation and the orchestration of the respiratory rhythm. Respir. Physiol. Neurobiol. 164, 96–104 10.1016/j.resp.2008.06.007 18602029 PMC3606077

[B17] Doi A. Ramirez J. M. (2010). State-dependent interactions between excitatory neuromodulators in the neuronal control of breathing. J. Neurosci. 30, 8251–8262 10.1523/JNEUROSCI.5361-09.2010 20554877 PMC3606074

[B18] Dutschmann M. Bischoff A. M. Büsselberg D. Richter D. W. (2003). Histaminergic modulation of the intact respiratory network of adult mice. Pflugers Arch. 445, 570–576 12634928 10.1007/s00424-002-0904-z

[B19] Errchidi S. Hilaire G. Monteau R. (1990). Permanent release of noradrenaline modulates respiratory frequency in the newborn rat, an in vitro study. J. Physiol. (Lond.) 429, 497–510 2277355 10.1113/jphysiol.1990.sp018269 PMC1181712

[B20] Farrant M. Nusser Z. (2005). Variations on an inhibitory theme, phasic and tonic activation of GABA A). receptors. Nat. Rev. Neurosci. 6, 215–229 10.1038/nrn1625 15738957

[B21] Feldman J. L. Del Negro C. A. (2006). Looking for inspiration, new perspectives on respiratory rhythm. Nat. Rev. Neurosci. 7, 232–242 10.1038/nrn1871 16495944 PMC2819067

[B22] Fewell J. E. Zhang C. Gillis A. M. (2007). Influence of adenosine A1-receptor blockade and vagotomy on the gasping and heart rate response to hypoxia in rats during early postnatal maturation. J. Appl. Physiol. 103, 1234–1241 10.1152/japplphysiol.01421.2006 17641210

[B23] Fujii M. Arata A. (2010). Adrenaline modulates on the respiratory network development. Adv. Exp. Med. Biol. 669, 25–28 10.1007/978-1-4419-5692-7_5 20217314

[B24] Funk G. D. Smith J. C. Feldman J. L. (1993). Generation and transmission of respiratory oscillations in medullary slices, role of excitatory amino acids. J. Neurophysiol. 70, 1497–1515 8283211 10.1152/jn.1993.70.4.1497

[B25] Gourine A. V. Kasymov V. Marina N. Tang F. Figueiredo M. F. Lane S. Teschemacher A. G. Spyer K. M. Deisseroth K. Kasparov S. (2010). Astrocytes control breathing through pH-dependent release of ATP. Science 329, 571–575 10.1126/science.1190721 20647426 PMC3160742

[B26] Gourine A. V. Llaudet E. Dale N. Spyer K. M. (2005). Release of ATP in the ventral medulla during hypoxia in rats, role in hypoxic ventilatory response. J. Neurosci. 25, 1211–1218 10.1523/JNEUROSCI.3763-04.2005 15689558 PMC6725960

[B27] Gozal D. Gozal E. Reeves S. R. Lipton A. J. (2002). Gasping and autoresuscitation in the developing rat, effect of antecedent intermittent hypoxia. J. Appl. Physiol. 92, 1141–1144 11842051 10.1152/japplphysiol.00972.2001

[B28] Greer J. J. Smith J. C. Feldman J. L. (1991). Role of excitatory amino acids in the generation and transmission of respiratory drive in neonatal rat. J. Physiol. (Lond.) 437, 727–749 1653855 10.1113/jphysiol.1991.sp018622 PMC1180074

[B29] Günther S. Maroteaux L. Schwarzacher S. W. (2006). Endogenous 5-HT2B receptor activation regulates neonatal respiratory activity in vitro. J. Neurobiol. 66, 949–961 10.1002/neu.20253 16758492

[B30] Härtel K. Schnell C. Hülsmann S. (2009). Astrocytic calcium signals induced by neuromodulators via functional metabotropic receptors in the ventral respiratory group of neonatal mice. Glia 57, 815–827 10.1002/glia.20808 19031447

[B31] Hehre D. A. Devia C. J. Bancalari E. Suguihara C. (2008). Brainstem amino acid neurotransmitters and ventilatory response to hypoxia in piglets. Pediatr. Res. 63, 46–50 10.1203/PDR.0b013e31815b4421 18043517

[B32] Herlenius E. Lagercrantz H. (1999). Adenosinergic modulation of respiratory neurons in the neonatal rat brainstem in vitro. J. Physiol. (Lond.) 518, 159–172 10.1111/j.1469-7793.1999.0159r.x 10373698 PMC2269420

[B33] Hilaire G. Viemari J. C. Coulon P. Simonneau M. Bévengut M. (2004). Modulation of the respiratory rhythm generator by the pontine noradrenergic A5 and A6 groups in rodents. Respir. Physiol. Neurobiol. 143, 187–197 10.1016/j.resp.2004.04.016 15519555

[B34] Hülsmann S. Oku Y. Zhang W. Richter D. W. (2000). Metabolic coupling between glia and neurons is necessary for maintaining respiratory activity in transverse medullary slices of neonatal mouse. Eur. J. Neurosci. 12, 856–862 10.1046/j.1460-9568.2000.00973.x 10762315

[B35] Huxtable A. G. Zwicker J. D. Alvares T. S. Ruangkittisakul A. Fang X. Hahn L. B. Posse de Chaves E. Baker G. B. Ballanyi K. Funk G. D. (2010). Glia contribute to the purinergic modulation of inspiratory rhythm-generating networks. J. Neurosci. 30, 3947–3958 10.1523/JNEUROSCI.6027-09.2010 20237265 PMC6632270

[B36] Huxtable A. G. Zwicker J. D. Poon B. Y. Pagliardini S. Vrouwe S. Q. Greer J. J. Funk G. D. (2009). Tripartite purinergic modulation of central respiratory networks during perinatal development, the influence of ATP., ectonucleotidases, and ATP metabolites. J. Neurosci. 29, 14713–14725 10.1523/JNEUROSCI.2660-09.2009 19940166 PMC6666021

[B37] Katz P. S. (1998). Comparison of extrinsic and intrinsic neuromodulation in two central pattern generator circuits in invertebrates. Exp. Physiol. 83, 281–292 9639339 10.1113/expphysiol.1998.sp004113

[B38] Kawai A. Okada Y. Mückenhoff K. Scheid P. (1995). Theophylline and hypoxic ventilator response in the rat isolated brainstem-spinal cord. Respir. Physiol. 100, 25–32 10.1016/0034-5687(94)00124-I 7604181

[B39] Kinney H. C. Filiano J. J. White W. F. (2001). Medullary serotonergic network deficiency in the sudden infant death syndrome, review of a 15-year study of a single dataset. J. Neuropathol. Exp. Neurol. 60, 228–247 11245208 10.1093/jnen/60.3.228

[B40] Kosofsky B. E. Molliver M. E. (1987). The serotoninergic innervation of cerebral cortex, different classes of axon terminals arise from dorsal and median raphe nuclei. Synapse 2, 153–168 10.1002/syn.890010204 2463687

[B41] Lalley P. M. (2004). Dopamine1 receptor agonists reverse opioid respiratory network depression, increase CO2 reactivity. Respir. Physiol. Neurobiol. 139, 247–262 10.1016/j.resp.2003.10.007 15122991

[B42] Lalley P. M. (2005). D1-dopamine receptor blockade slows respiratory rhythm and enhances opioid-mediated depression. Respir. Physiol. Neurobiol. 145, 13–22 10.1016/j.resp.2004.11.001 15652784

[B43] Lambe E. K. Aghajanian G. K. (2006). Hallucinogen-induced UP states in the brain slice of rat prefrontal cortex, role of glutamate spillover and NR2B-NMDA receptors. Neuropsychopharmacology 31, 1682–1689 10.1038/sj.npp.1300944 16292328

[B44] Lambe E. K. Aghajanian G. K. (2007). Prefrontal cortical network activity, opposite effects of psychedelic hallucinogens and D1/D5 dopamine receptor activation. Neuroscience 145, 900–910 10.1016/j.neuroscience.2006.12.048 17293055 PMC1894690

[B45] Lawson E. E. Waldrop T. G. Eldridge F. L. (1979). Naloxone enhances respiratory output in cats. J. Appl. Physiol. 47, 1105–1111 511712 10.1152/jappl.1979.47.5.1105

[B46] Lieske S. P. Thoby-Brisson M. Telgkamp P. Ramirez J. M. (2000). Reconfiguration of the neural network controlling multiple breathing patterns, eupnea, sighs and gasps. Nat. Neurosci. 3, 600–607 10.1038/75776 10816317

[B47] Liu Q. Wong-Riley M. T. (2010). Postnatal development of N-methyl-D-aspartate receptor subunits 2A, 2B, 2C, 2D, and 3B immunoreactivity in brain stem respiratory nuclei of the rat. Neuroscience 171, 637–654 10.1016/j.neuroscience.2010.09.052 20887777 PMC2987514

[B48] Lorier A. R. Huxtable A. G. Robinson D. M. Lipski J. Housley G. D. Funk G. D. (2007). P2Y1 receptor modulation of the pre-Bötzinger complex inspiratory rhythm generating network in vitro. J. Neurosci. 27, 993–1005 10.1523/JNEUROSCI.3948-06.2007 17267553 PMC6673186

[B49] Lorier A. R. Lipski J. Housley G. D. Greer J. J. Funk G. D. (2008). ATP sensitivity of preBötzinger complex neurones in neonatal rat in vitro, mechanism underlying a P2 receptor-mediated increase in inspiratory frequency. J. Physiol. (Lond.) 586, 1429–1446 10.1113/jphysiol.2007.143024 18174215 PMC2375674

[B50] Mann E. O. Mody I. (2010). Control of hippocampal gamma oscillation frequency by tonic inhibition and excitation of interneurons. Nat. Neurosci. 13, 205–212 10.1038/nn.2464 20023655 PMC2843436

[B51] Marder E. Bucher D. (2007). Understanding circuit dynamics using the stomatogastric nervous system of lobsters and crabs. Annu. Rev. Physiol. 69, 291–316 10.1146/annurev.physiol.69.031905.161516 17009928

[B52] Martín E. D. Fernández M. Perea G. Pascual O. Haydon P. G. Araque A. Ceña V. (2007). Adenosine released by astrocytes contributes to hypoxia-induced modulation of synaptic transmission. Glia 55, 36–45 10.1002/glia.20431 17004232

[B53] Mathew O. P. (2011). Apnea of prematurity, pathogenesis, and management strategies. J. Perinatol. 31, 302–310 10.1038/jp.2010.126 21127467

[B54] Morgado-Valle C. Feldman J. L. (2004). Depletion of substance P and glutamate by capsaicin blocks respiratory rhythm in neonatal rat in vitro. J. Physiol. (Lond.) 555, 783–792 10.1113/jphysiol.2003.060350 14724197 PMC1664860

[B55] Morgado-Valle C. Feldman J. L. (2007). NMDA receptors in preBotzinger complex neurons can drive respiratory rhythm independent of AMPA receptors. J. Physiol. (Lond.) 582, 359–368 10.1113/jphysiol.2007.130617 17446224 PMC2075291

[B56] Mutolo D. Cinelli E. Bongianni F. Pantaleo T. (2011). Identification of a cholinergic modulatory and rhythmogenic mechanism within the lamprey respiratory network. J. Neurosci. 31, 13323–13332 10.1523/JNEUROSCI.2764-11.2011 21917815 PMC6623278

[B57] Nusser Z. Sieghart W. Somogyi P. (1998). Segregation of different GABAA receptors to synaptic, and extrasynaptic membranes of cerebellar granule cells. J. Neurosci. 18, 1693–1703 9464994 10.1523/JNEUROSCI.18-05-01693.1998 PMC6792611

[B58] Paarmann I. Frermann D. Keller B. U. Hollmann M. (2000). Expression of 15 glutamate receptor subunits and various splice variants in tissue slices and single neurons of brainstem nuclei and potential functional implications. J. Neurochem. 74, 1335–1345 10.1046/j.1471-4159.2000.0741335.x 10737588

[B59] Paarmann I. Frermann D. Keller B. U. Villmann C. Breitinger H. G. Hollmann M. (2005). Kinetics and subunit composition of NMDA receptors in respiratory-related neurons. J. Neurochem. 93, 812–824 10.1111/j.1471-4159.2005.03027.x 15857385

[B60] Papatheodoropoulos C. Koniaris E. (2011). α5GABAA receptors regulate hippocampal sharp wave-ripple activity in vitro. Neuropharmacology 60, 662–673 10.1016/j.neuropharm.2010.11.022 21146551

[B61] Peña F. (2008). Contribution of pacemaker neurons to respiratory rhythms generation in vitro. Adv. Exp. Med. Biol. 605, 114–118 10.1007/978-0-387-73693-8_20 18085257

[B62] Peña F. (2009). Neuronal network properties underlying the generation of gasping. Clin. Exp. Pharmacol. Physiol. 36, 1218–1228 10.1111/j.1440-1681.2009.05301.x 19793109

[B63] Peña F. Aguileta M. A. (2007). Effects of riluzole, and flufenamic acid on eupnea, and gasping of neonatal mice in vivo. Neurosci. Lett. 415, 288–293 10.1016/j.neulet.2007.01.032 17276002

[B64] Peña F. García O. (2006). Breathing generation and potential pharmacotherapeutic approaches to central respiratory disorders. Curr. Med. Chem. 13, 2681–2693 10.2174/092986706778201602 17017919

[B65] Peña F. Meza-Andrade R. Páez-Zayas V. González-Marín M. C. (2008). Gasping generation in developing Swiss-Webster mice in vitro and in vivo. Neurochem. Res. 33, 1492–1500 10.1007/s11064-008-9616-x 18273701

[B66] Peña F. Parkis M. A. Tryba A. K. Ramirez J. M. (2004). Differential contribution of pacemaker properties to the generation of respiratory rhythms during normoxia, and hypoxia. Neuron 43, 105–117 10.1016/j.neuron.2004.06.023 15233921

[B67] Peña F. Ramirez J. M. (2002). Endogenous activation of serotonin-2A receptors is required for rhythmogenesis in vitro. J. Neurosci. 22, 11055–11064 12486201 10.1523/JNEUROSCI.22-24-11055.2002 PMC6758407

[B68] Peña F. Ramirez J. M. (2004). Substance P-mediated modulation of pacemaker properties in the mammalian respiratory network. J. Neurosci. 24, 7549–7556 10.1523/JNEUROSCI.2924-04.2004 15329402 PMC6729648

[B69] Peña F. Ramirez J. M. (2005). Hypoxia-induced changes in neuronal network properties. Mol. Neurobiol. 32, 251–283 10.1385/MN:32:3:251 16385141

[B70] Peña F. Tapia R. (1999). Relationships among seizures, extracellular amino acid changes, and neurodegeneration induced by 4-aminopyridine in rat hippocampus: a microdialysis and electrncephalographic study. J. Neurochem. 72, 2006–2014 10.1046/j.1471-4159.1999.0722006.x 10217278

[B71] Peña F. Tapia R. (2000). Seizures and neurodegeneration induced by 4-aminopyridine in rat hippocampus in vivo: role of glutamate- and GABA-mediated neurotransmission and of ion channels. Neuroscience 101, 547–561 10.1016/S0306-4522(00)00400-0 11113304

[B72] Poets C. F. Meny R. G. Chobanian M. R. Bonofiglo R. E. (1999). Gasping and other cardiorespiratory patterns during sudden infant deaths. Pediatr. Res. 45, 350–354 10.1203/00006450-199904020-02082 10088653

[B73] Ptak K. Yamanishi T. Aungst J. Milescu L. S. Zhang R. Richerson G. B. Smith J. C. (2009). Raphé neurons stimulate respiratory circuit activity by multiple mechanisms via endogenously released serotonin and substance P. J. Neurosci. 29, 3720–3737 10.1523/JNEUROSCI.5271-08.2009 19321769 PMC2940110

[B74] Ramirez J. M. Schwarzacher S. W. Pierrefiche O. Olivera B. M. Richter D. W. (1998). Selective lesioning of the cat pre-Bötzinger complex in vivo eliminates breathing but not gasping. J. Physiol. (Lond.) 507, 895–907 10.1111/j.1469-7793.1998.571bt.x 9508848 PMC2230836

[B75] Ramirez J. M. Tryba A. K. Peña F. (2004). Pacemaker neurons and neuronal networks, an integrative view. Curr. Opin. Neurobiol. 14, 665–674 10.1016/j.conb.2004.10.011 15582367

[B76] Ramírez-Jarquín J. O. Lara-Hernández S. López-Guerrero J. J. Aguileta M. A. Rivera-Angulo A. J. Sampieri A. Vaca L. Ordaz B. Peña-Ortega F. (2012). Somatostatin modulates generation of inspiratory rhythms and determines asphyxia survival. Peptides 34, 360–372 10.1016/j.peptides.2012.02.011 22386651

[B77] Ren J. Greer J. J. (2006). Neurosteroid modulation of respiratory rhythm in rats during the perinatal period. J. Physiol. (Lond.) 574, 535–546 10.1113/jphysiol.2006.108829 16690709 PMC1817773

[B78] Richerson G. B. (2004). Serotonergic neurons as carbon dioxide sensors that maintain pH homeostasis. Nat. Rev. Neurosci. 5, 449–461 10.1038/nrn1409 15152195

[B79] Richter D. W. Schmidt-Garcon P. Pierrefiche O. Bischoff A. M. Lalley P. M. (1999). Neurotransmitters and neuromodulators controlling the hypoxic respiratory response in anaesthetized cats. J. Physiol. (Lond.) 514, 567–578 10.1111/j.1469-7793.1999.567ae.x 9852336 PMC2269078

[B80] Scanziani M. (2000). GABA spillover activates postsynaptic GABA B) receptors to control rhythmic hippocampal activity. Neuron 25, 673–681 10.1016/S0896-6273(00)81069-7 10774734

[B81] Schlenker E. H. Inamdar S. R. (1995). Effects of naloxone on oxygen consumption and ventilation in awake golden Syrian hamsters. Physiol. Behav. 57, 655–658 10.1016/0031-9384(94)00297-5 7777599

[B82] Schmidt C. Bellingham M. C. Richter D. W. (1995). Adenosinergic modulation of respiratory neurones and hypoxic responses in the anaesthetized cat. J. Physiol. (Lond.) 483, 769–781 7776257 10.1113/jphysiol.1995.sp020621 PMC1157817

[B83] Schnell C. Fresemann J. Hülsmann S. (2011). Determinants of functional coupling between astrocytes and respiratory neurons in the pre-Bötzinger complex. PLoS ONE 6, e26309 10.1371/journal.pone.0026309 22039458 PMC3198395

[B84] Schwarzacher S. W. Pestean A. Günther S. Ballanyi K. (2002). Serotonergic modulation of respiratory motoneurons and interneurons in brainstem slices of perinatal rats. Neuroscience 115, 1247–1259 10.1016/S0306-4522(02)00540-7 12453495

[B85] Schwarzacher S. W. Rüb U. Deller T. (2011). Neuroanatomical characteristics of the human pre-Bötzinger complex and its involvement in neurodegenerative brainstem diseases. Brain 134, 24–35 10.1093/brain/awq327 21115469

[B86] Scimemi A. Andersson A. Heeroma J. H. Strandberg J. Rydenhag B. McEvoy A. W. Thom M. Asztely F. Walker M. C. (2006). Tonic GABAA receptor-mediated currents in human brain. Eur. J. Neurosci. 24, 1157–1160 10.1111/j.1460-9568.2006.04989.x 16930441

[B87] Semyanov A. Walker M. C. Kullmann D. M. Silver R. A. (2004). Tonically active GABA A receptors, modulating gain and maintaining the tone. Trends Neurosci. 27, 262–269 10.1016/j.tins.2004.03.005 15111008

[B88] Shao X. M. Feldman J. L. (1997). Rhythmogenesis and synaptic inhibition of expiratory neurons in pre-Bötzinger complex, differential roles of glycinergic and GABAergic neural transmission. J. Neurophysiol. 77, 1853–1860 9114241 10.1152/jn.1997.77.4.1853

[B89] Shao X. M. Feldman J. L. (2005). Cholinergic neurotransmission in the preBötzinger Complex modulates excitability of inspiratory neurons and regulates respiratory rhythm. Neuroscience 130, 1069–1081 10.1016/j.neuroscience.2004.10.028 15653001 PMC4342058

[B90] Shao X. M. Feldman J. L. (2009). Central cholinergic regulation of respiration, nicotinic receptors. Acta Pharmacol. Sin. 30, 761–770 10.1038/aps.2009.88 19498418 PMC4002383

[B91] Shao X. M. Tan W. Xiu J. Puskar N. Fonck C. Lester H. A. Feldman J. L. (2008). Alpha4* nicotinic receptors in preBotzinger complex mediate cholinergic/nicotinic modulation of respiratory rhythm. J. Neurosci. 28, 519–528 10.1523/JNEUROSCI.3666-07.2008 18184794 PMC3477875

[B92] Smith J. C. Ellenberger H. H. Ballanyi K. Richter D. W. Feldman J. L. (1991). Pre-Bötzinger complex: a brainstem region that may generate respiratory rhythm in mammals. Science 254, 726–729 10.1126/science.1962207 1683005 PMC3209964

[B93] Sridhar R. Thach B. T. Kelly D. H. Henslee J. A. (2003). Characterization of successful and failed autoresuscitation in human infants, including those dying of SIDS. Pediatr. Pulmonol. 36, 113–122 10.1002/ppul.10287 12833490

[B94] Stell B. M. Brickley S. G. Tang C. Y. Farrant M. Mody I. (2003). Neuroactive steroids reduce neuronal excitability by selectively enhancing tonic inhibition mediated by delta subunit-containing GABAA receptors. Proc. Natl. Acad. Sci. U.S.A. 100, 14439–14444 10.1073/pnas.2435457100 14623958 PMC283610

[B95] Telgkamp P. Cao Y. Q. Basbaum A. I. Ramirez J. M. (2002). Long-term deprivation of substance P in PPT-A mutant mice alters the anoxic response of the isolated respiratory network. J. Neurophysiol. 88, 206–213 12091546 10.1152/jn.2002.88.1.206

[B96] Thoby-Brisson M. Ramirez J. M. (2001). Identification of two types of inspiratory pacemaker neurons in the isolated respiratory neural network of mice. J. Neurophysiol. 86, 104–112 11431492 10.1152/jn.2001.86.1.104

[B97] Torrealba F. Carrasco M. A. (2004). A review on electron microscopy and neurotransmitter systems. Brain Res. Brain Res. Rev. 47, 5–17 10.1016/j.brainresrev.2004.06.004 15572159

[B98] Towers S. K. Gloveli T. Traub R. D. Driver J. E. Engel D. Fradley R. Rosahl T. W. Maubach K. Buhl E. H. Whittington M. A. (2004). Alpha 5 subunit-containing GABAA receptors affect the dynamic range of mouse hippocampal kainate-induced gamma frequency oscillations in vitro. J. Physiol. (Lond.) 559, 721–728 15284346 10.1113/jphysiol.2004.071191 PMC1665170

[B99] Tryba A. K. Peña F. Ramirez J. M. (2006). Gasping activity in vitro, a rhythm dependent on 5-HT2A receptors. J. Neurosci. 26, 2623–2634 10.1523/JNEUROSCI.4186-05.2006 16525041 PMC6675157

[B100] Viemari J. C. (2008). Noradrenergic modulation of the respiratory neural network. Respir. Physiol. Neurobiol. 164, 123–130 10.1016/j.resp.2008.06.016 18634907

[B101] Viemari J. C. Garcia A. J. III Doi A. Ramirez J. M. (2011). Activation of alpha-2 noradrenergic receptors is critical for the generation of fictive eupnea and fictive gasping inspiratory activities in mammals in vitro. Eur. J. Neurosci. 33, 2228–2237 10.1111/j.1460-9568.2011.07706.x 21615559 PMC3652413

[B102] Vizi E. S. Fekete A. Karoly R. Mike A. (2010). Non-synaptic receptors and transporters involved in brain functions and targets of drug treatment. Br. J. Pharmacol. 160, 785–809 10.1111/j.1476-5381.2009.00624.x 20136842 PMC2935987

[B103] Watanabe M. Mishina M. Inoue Y. (1994). Distinct distributions of five NMDA receptor channel subunit mRNAs in the brainstem. J. Comp. Neurol. 343, 520–531 10.1002/cne.903430403 7518475

[B104] Wenninger J. M. Pan L. G. Klum L. Leekley T. Bastastic J. Hodges M. R. Feroah T. R. Davis S. Forster H. V. (2004). Large lesions in the pre-Bötzinger complex area eliminate eupneic respiratory rhythm in awake goats. J. Appl. Physiol. 97, 1629–1636 10.1152/japplphysiol.00953.2003 15247161

[B105] Wilken B. Ramirez J. M. Hanefeld F. Richter D. W. (2000). Aminophylline modulation of the mouse respiratory network changes during postnatal maturation. J. Appl. Physiol. 89, 2015–2022 11053357 10.1152/jappl.2000.89.5.2015

[B106] Wotjak C. T. Landgraf R. Engelmann M. (2008). Listening to neuropeptides by microdialysis, echoes and new sounds? Pharmacol. Biochem. Behav. 90, 125–134 10.1016/j.pbb.2008.03.017 18468668

[B107] Young J. K. Dreshaj I. A. Wilson C. G. Martin R. J. Zaidi S. I. Haxhiu M. A. (2005). An astrocyte toxin influences the pattern of breathing and the ventilatory response to hypercapnia in neonatal rats. Respir. Physiol. Neurobiol. 147, 19–30 10.1016/j.resp.2005.01.009 15848120

[B108] Zanella S. Roux J. C. Viemari J. C. Hilaire G. (2006). Possible modulation of the mouse respiratory rhythm generator by A1/C1 neurones. Respir. Physiol. Neurobiol. 153, 126–138 10.1016/j.resp.2005.09.009 16309976

[B109] Zavala-Tecuapetla C. Aguileta M. A. Lopez-Guerrero J. J. González-Marín M. C. Peña F. (2008). Calcium-activated potassium currents differentially modulate rhythmogenesis. Eur. J. Neurosci. 27, 2871–2884 10.1111/j.1460-9568.2008.06214.x 18445052

[B110] Zwicker J. D. Rajani V. Hahn L. B. Funk G. D. (2011). Purinergic modulation of preBötzinger complex inspiratory rhythm in rodents: the interaction between ATP and adenosine. J. Physiol. (Lond.) 589, 4583–4600 21788352 10.1113/jphysiol.2011.210930 PMC3208226

